# Retroperitoneoscopic pyeloplasty with simultaneous pyelolithotomy using a flexible cystoscope: Our initial experience at a single centre

**DOI:** 10.3389/fsurg.2022.938911

**Published:** 2022-08-19

**Authors:** Fei Zhang, Li Wang, ZheBin Gao, HouMeng Yang

**Affiliations:** Department of Urology, Hwa Mei Hospital, University of Chinese Academy of Sciences (Ningbo No.2 Hospital), Ningbo, China

**Keywords:** ureteropelvic junction obstruction, flexible cystoscope, kidney stone, retroperitoneoscopy, pyeloplasty

## Abstract

**Purpose:**

We present our experience with retroperitoneoscopic pyeloplasty with simultaneous pyelolithotomy using a flexible cystoscope in patients with ureteropelvic junction obstruction(UPJO) complicated with kidney stones.

**Materials and Methods:**

The records of 37 patients who underwent retroperitoneoscopic pyeloplasty with simultaneous pyelolithotomy using a flexible cystoscope to manage UPJO complicated with kidney stones from July 2015 to December 2020 were retrospectively reviewed. All patients underwent one-stage retroperitoneoscopic pyeloplasty combined with flexible cystoscopic pyelolithotomy. The operative time, blood volume, stone clearance rate, length of hospital stay, complications and follow-up events were recorded.

**Results:**

The operation went smoothly in all 37 patients. The mean operative time was 148.4 ± 24.2 min. The mean intraoperative blood loss volume was 54.3 ± 20.5 ml. The mean hospitalization time was 10.6 ± 3.7 days. The stone clearance rate was 81.08%. The mean follow-up period was 23.5 months (range 12–53 months). Hydronephrosis was significantly decreased in 33 of the 37 cases. The success rate of the operation was 89.19%. Stones recurred in 9 patients during follow-up, for a recurrence rate of 24.32%.

**Conclusion:**

Retroperitoneoscopic pyeloplasty with simultaneous pyelolithotomy using a flexible cystoscope in patients with UPJO complicated with kidney stones is safe, effective and worthy of promotion.

## Introduction

Ureteropelvic junction obstruction is a common anatomic lesion in urology. Its congenital incidence is approximately 0.1% (1). UPJO is complicated with kidney stones in up to 20% of cases due to obstruction and hydronephrosis (2, 3). UPJO complicated with kidney stones is difficult to treat surgically. Surgical methods for treating UPJO complicated by kidney stones include open surgery, percutaneous nephrolithotomy (PNL) with endopyelotomy, retroperitoneoscopic or laparoscopic robot-assisted pyeloplasty with flexible or rigid scopes, and simultaneous or staged surgery (4, 5). In the past, PNL with endopyelotomy was the preferred treatment, but its long-term efficacy is unsatisfactory (6). Staged surgery adds time and financial cost to patients. For most hospitals and patients in China, the Da Vinci surgical robotic system (DVSS) is too expensive (7). In recent years, with the rapid development of laparoscopic and endoscopic surgery, combined laparoscopic and endoscopic technology in the management of UPJO complicated with kidney stones has achieved desirable results (8–10).

From July 2015 to December 2020, a retroperitoneoscopic method combined with the use of a flexible cystoscope was used for pyelolithotomy in 37 cases in our hospital, and it achieved favourable efficacy. The present study aimed to introduce our experience with combined retroperitoneoscopy and flexible cystoscopic pyelolithotomy for patients with UPJO complicated with kidney stones.

## Materials and methods

### Clinical data

The study was approved by the ethics committee of our hospital. Written informed consent was obtained from all patients. All patients underwent intravenous pyelography (IVP) and CT examinations routinely. If IVP imaging was not clear, retrograde pyelography was performed. Inclusion criteria were a diagnosis of UPJO complicated with kidney stones and treatment with retroperitoneoscopic pyeloplasty with simultaneous pyelolithotomy using a flexible cystoscope. Patients who were complicated with a nonfunctional kidney were excluded from our analysis. From July 2015 to May 2020, 37 patients with symptomatic ureteropelvic obstruction and kidney stones underwent retroperitoneoscopic pyeloplasty with concomitant pyelolithotomy. The age range of the participants was 27–67 years, and the mean age was 43.8 ± 11.1 years. The clinical manifestations included back pain in 29 patients, gross haematuria in 11 patients, and recurrent pyelonephritis in 10 patients. Mild hydronephrosis, moderate hydronephrosis and severe hydronephrosis were diagnosed in 3 cases, 25 cases and 9 cases, respectively. Seven patients had a single kidney stone, and 30 patients had multiple kidney stones, with a maximum diameter of 23 mm. Eight patients received other treatments prior to admission, such as high-pressure balloon catheter dilation or ureteral stent placement. Enhanced urinary computed tomography was performed preoperatively for all patients, and retrograde renal pyelography was employed for 9 patients to clarify the diagnosis. Other clinical data were shown in Table 1.

**Table 1:** Demographic characteristics and surgical statistics.

Variable	
Sex (*n*)	
Male	24 (64.86%)
Female	13 (35.14%)
Age (years)	43.8 ± 11.1
20–40	13 (35.14%)
40–60	19 (51.35%)
>60	5 (13.51%)
Renal calculus (n)	
Multiple	30 (81.08%)
Solitary	7 (18.92%)
Surgical spot (n)	
Left	20 (54.05%)
Right	17 (45.95%)
Symptoms (n)	
Lumbago	29 (78.38%)
Pyelonephritis	10 (27.03%)
Gross haematuria	11 (29.73%)
Hydronephrosis (n)	
Mild	3 (8.11%)
Moderate	25 (67.57%)
Severe	9 (24.32%)
Previous other treatments (n)	8 (21.62%)
Operative time (min)	148.4 ± 24.2
60–120	5 (13.51%)
120–180	23 (62.16%)
>180	9 (24.32%)
Blood loss volume (ml)	54.3 ± 20.5
0–50	15 (40.54%)
50–100	18 (48.65%)
>100	4 (10.81%)

### Operative method

Two sets of endoscopic monitoring equipment were placed at the head of the patient. After general anaesthesia was induced, an indwelling catheter was placed. Then, the patient was placed in the flank position, depending upon the side to be operated on.

**Figure 1:**
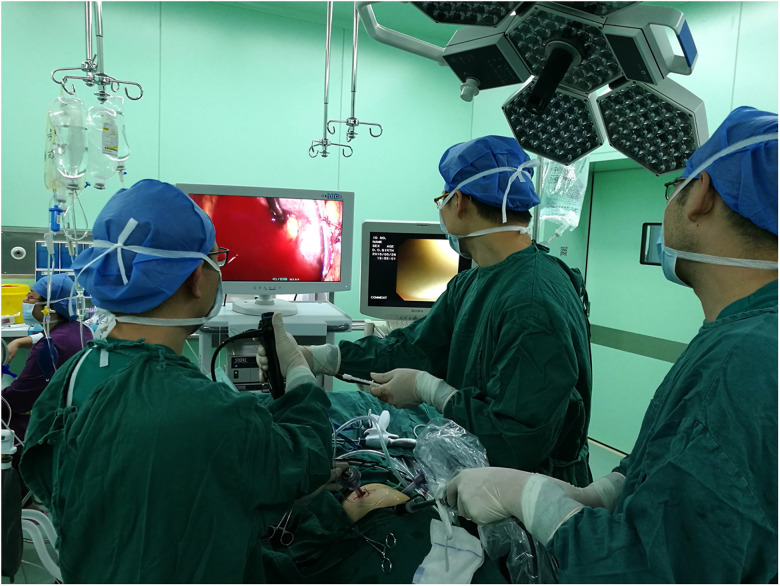
(**A**) Two monitors were placed at the head of the patient. The surgeon stood behind the patient, and the assistant stood in front of the patient. (**B**) The flexible cystoscope was placed in the retroperitoneal space through a 12 mm trocar and was then inserted into the pelvis to detect the stone with the aid of the assistant. (**C**) The fragments were extracted using a Nitinol stone basket.

The primary port, a 12 mm incision, was made 1 cm below the 12th rib margin of the posterior axillary line. Balloon dilation through this port was done to create the retroperitoneal space. Once balloon dilation was completed, the second port, a 12 mm incision, was placed 1 cm below the 12th rib margin of the anterior axillary line. The third port, a 12 mm incision, was made at the midaxillary line approximately two finger breadths cephalad to the anterior superior iliac spine. The third port was used to hold the laparoscope. Antibiotics were administered during anaesthesia induction. The perirenal fascia was longitudinally opened. The inferior pole of the kidney was dissociated. The renal pelvis and upper ureter were exposed. A 15 mm incision was made in the pelvis. The flexible cystoscope was placed into the retroperitoneal space through a 12 mm trocar and then inserted into the pelvis to detect the stone with the aid of the assistant (Figure 1B). The stone was removed using a stone basket catheter through the working channel of the flexible cystoscope (Figure 1C). The stone was ablated using a holmium laser if it was too large to be removed. Whether the stone had been completely removed was verified by referring to the preoperative computed tomography scan. The ureteropelvic junction obstruction was managed on the basis of the Anderson-Hynes procedure. At the end of the operation, an F6 double-J stent was placed into the ureter. Urological computed tomography examination was performed on the third day after the operation, and the ureteral stent was removed two months after the operation. CT urography was carried out three months after the surgery. Successful operation was defined as a CT reexamination showing a significant reduction in hydronephrosis after the removal of the double J tubes. Urological ultrasonography examination was performed every 3 months.

## Results

The operation went smoothly in all 37 patients. The mean operative time was 148.4 ± 24.2 min (range, 97–182). The mean intraoperative blood loss volume was 54.3 ± 20.5 ml (range, 25–150). The mean hospitalization time was 10.6 ± 3.7 days (range 7–11). Six patients suffered from complications (Clavien I and IIIa). Three patients had postoperative fever, which was treated medically. Three patients suffered urine leakage from the double-J stent bending; the perirenal effusion was absorbed two weeks later after repositioning of the double-J stent. At two months, 7 patients had residual calculi, for a stone clearance rate of 81.08%. Consequently, these patients underwent further stone management, including ureteroscopy and shock wave lithotripsy. The mean follow-up period was 23.5 months (range 12–53 months). Frequent haematuria occurred in 8 patients before removal of ureteral stents, and 11 patients presented with mild back pain. Hydronephrosis was significantly decreased in 33 of the 37 patients, for a success rate of the UPJO operation of 89.19%. Stones recurred in 9 patients during follow-up, a recurrence rate of 24.32% (Table 2).

**Table 2:** Complications and follow-up.

Complications (Clavien I-II) (n)	
Hospital complications(n)	6 (16.22%)
Urine leak	3 (8.11%)
Fever	3 (8.11%)
Out-of-hospital complications(n)	
Haematuria	8 (21.62%)
Backache	7 (18.92%)
Follow up	
Stone-free rate	81.08% (30/37)
Success rate	89.19% (33/37)
Recurrence rate of stones	24.32% (9/37)

## Discussion

UPJO is a common anatomic lesion in urology. Its congenital incidence is approximately 0.1%. Up to 20% of UPJOs are complicated with kidney stones due to obstruction and hydronephrosis. In the past 20 years, with the development of laparoscopy, laparoscopic pyeloplasty has become the main treatment for UPJO. The laparoscopic technique is superior to open surgery due to its low trauma, favourable field of exposure and ease of suturing (11).

In patients diagnosed with UPJO complicated with kidney stones, the stones tend to be located in the lower calyx of the kidney. The treatment of UPJO complicated with kidney stones with a laparoscopic method alone is frequently not desirable. When percutaneous lithotripsy has been performed to treat both UPJO and kidney stones, the postoperative follow-up indicated poor outcomes (4–6). A two-stage procedure for patients with UPJO complicated with kidney stones, including a first-stage laparoscopic pyeloplasty for UPJO and second-stage percutaneous nephroscopy for kidney stones, is effective, but this increases the medical costs to the patient significantly.

The angle of the renal pelvis and lower calyx make it very difficult to deal with kidney stones. The treatment of UPJO combined with kidney stones using a laparoscopic or endoscopic method alone is difficult and unsatisfactory. In recent years, some scholars have reported that the combination of laparoscopic or robotic laparoscopic surgery and flexible cystoscopy in the treatment of UPJO complicated with kidney stones leads to excellent outcomes in terms of both the stone clearance rate and protecting renal function. Peng et al. introduced a flexible guiding tube as a simple modification of laparoscopic pyeloplasty combined with a flexible cystoscope for the treatment of UPJO complicated with renal calculi (12). An et al. introduced laparoscopic pyeloplasty combined with a 19.5-F rigid nephroscope for the treatment of UPJO complicated with kidney stones (13). Lambertini et al. reported on 43 cases of UPJO with urolithiasis that were treated with robotic-assisted pyeloplasty with endoscopic removal of stones, and the effects were satisfactory (14). Hüttenbrink et al. reported a stone clearance rate of 100% with no complications from a combination of robotic pyeloplasty and percutaneous renal surgery for the treatment of UPJO and calyx stones (15).

Since July 2015, we have used retroperitoneoscopic pyeloplasty combined with antegrade flexible cystoscopic pyelolithotomy in one stage to treat 37 cases of UPJO complicated with kidney stones, with a stone clearance rate of 81.08% and a UPJO operation success rate of 89.19%. The advantages of our procedure include the following: (1) When UPJO is complicated with kidney stones, most of the stones are located in the lower renal calyx, and the angle of the renal pelvis and lower calyx makes it very difficult to deal with kidney stones there. However, in anterograde flexible cystoscopic pyelolithotomy, the stone clearance rate is almost unaffected by the angle. (2) The body of the flexible cystoscope used for pyelolithotomy is thick and short, making it more manipulable through anterograde procedures than a flexible ureteroscope. However, for patients with a narrow calyceal neck, an antegrade flexible ureteroscope can be used to remove the stone. Of the 37 cases here, 6 needed the flexible ureteroscope due to a narrow calyx neck, while the remaining procedures were completed by flexible cystoscopic pyelolithotomy. (3) A 15 mm incision in the renal pelvis is a good size. A smaller incision causes difficulty removing stones, and larger incisions cause difficulties in filling the renal pelvis with perfusion fluid, leading to renal pelvis collapse and no room for the operation. During flexible cystoscopic pyelolithotomy, perfusion fluid in the renal pelvis is removed through a suction apparatus by an assistant to help keep the surgical field clear. (4) Patience and carefulness are indispensable for performing antegrade flexible cystoscopic pyelolithotomy due to changes in operating habits. Larger kidney stones can be broken into pieces with a holmium laser, followed by removal with a stone basket. (5) The retroperitoneal approach is as effective as the intraperitoneal approach, with little interference by abdominal organs and rapid postoperative recovery. Sometimes, operation through the intraperitoneal approach may cause abdominal infection and intestinal adhesion. (6) Two procedures, retroperitoneoscopic pyeloplasty and flexible cystoscopic pyelolithotomy, can complement each other to combine their advantages and avoid their disadvantages and clear stones to the greatest extent. (7) Retroperitoneoscopic pyeloplasty and flexible cystoscopic pyelolithotomy can be used to manage UPJO complicated with kidney stones in one stage, thus avoiding a second surgery and reducing medical costs.

Retroperitoneoscopic pyeloplasty combined with antegrade flexible cystoscopic pyelolithotomy for UPJO complicated with kidney stones is advantageous in terms of minimal trauma, rapid recovery and a high stone clearance rate. Retroperitoneoscopic pyeloplasty and antegrade flexible cystoscopic pyelolithotomy can complement each other. This technology can avoid a second operation, reduce the medical costs to patients, and efficiently treat UPJO complicated with kidney stones. Of course, there are some limitations to our work. Due to the lack of a DVSS here, we were unable to compare the pros and cons of DVSS and retroperitoneoscopy in the management of UPJO complicated with kidney stones, which may be a more interesting question. Though it is the most sensitive indicator of renal function, we rarely do kidney scans for a variety of reasons, and this might affect the quality of our conclusions. We look forward to conducting a larger, prospective, multicentre, well-equipped clinical trial to further investigate the treatment of UPJO complicated with kidney stones.

## Data Availability

The raw data supporting the conclusions of this article will be made available by the authors, without undue reservation.
